# MBI-KG: A knowledge graph of structured and linked economic research data extracted from the 1937 book “Die Maschinen-Industrie im Deutschen Reich”

**DOI:** 10.1016/j.dib.2024.111238

**Published:** 2024-12-17

**Authors:** Renat Shigapov, Thomas Schmidt, Jan Kamlah, Irene Schumm, Jochen Streb, Sibylle Lehmann-Hasemeyer

**Affiliations:** a University Library, University of Mannheim, Mannheim, Germany; b Department of Economics, University of Mannheim, Mannheim, Germany; c Faculty of Business, Economics and Social Sciences, University of Hohenheim, Stuttgart, Germany

**Keywords:** German company data, Mechanical engineering industry

## Abstract

The MaschinenBauIndustrie Knowledge Graph (MBI-KG) is a structured and semantically enriched dataset extracted from the 1937 publication “Die Maschinen-Industrie im Deutschen Reich” (The Machinery Industry in the German Reich), published by the “Wirtschaftsgruppe Maschinenbau” and edited by Herbert Patschan. This historical source offers data on German companies within the mechanical engineering industry during the pre-World War II era.

The book was digitized, and Optical Character Recognition (OCR) was applied to extract text. The unstructured extracted data was then structured and semantically enriched to enable data integration and reuse. The semantically enriched data was uploaded into an open-source knowledge-graph software. The resulting knowledge graph includes detailed information about companies, individuals, and administrative entities relevant to the German mechanical engineering industry. The data is accessible through various means, including a SPARQL endpoint, an API, advanced search functionalities, a reconciliation API, and bulk files. Each entity in the knowledge graph can be exported in multiple formats, such as CSV, RDF (ttl), JSON, and NDJSON, ensuring compatibility with diverse research tools and platforms.

This dataset can be reused in various research domains, including economic history, data science, and digital humanities. By providing machine-readable, structured data from a crucial historical period, the MBI-KG facilitates novel analyses and insights into the economic and industrial landscape of early 20th-century Germany. The dataset's interoperability with other data sources and its alignment with FAIR principles further enhance its value for interdisciplinary research and long-term preservation.

Specifications Table

**Table:** 

Subject	Economics
Specific subject area	Historical Economics
Type of data	Table, Graph, Processed
Data collection	The data [1] was collected by digitizing the 1937 book "Die Maschinen-Industrie im Deutschen Reich" [2] using a high-resolution scanner (book2net ultra by Microbox) in 400 dpi resolution. The transcription platform eScriptorium [3] (powered by the OCR engine Kraken [4]) was used to create layout segmentation information and full texts from these digital images. To ensure the quality of the research data, ground truth data for layout segmentation and text recognition was generated in an iterative approach to fine-tune existing segmentation and transcription models. The resulting transcriptions were then processed using the custom script book2entities based on the Python tool “blatt” [5], which parsed the unstructured text data and converted them into a structured format*.*
Data source location	Mannheim
Data accessibility	Repository name: MADATA (Mannheim Data Repository) Data identification number: https://doi.org/10.7801/467 Direct URL to data: https://madata.bib.uni-mannheim.de/467, https://github.com/UB-Mannheim/MBI-KG Instructions for accessing these data: 1) Download the file MBI-KG.zip archived at https://doi.org/10.7801/467. 2) Unzip the file. 3) The folder “data” contains the datasets created during this project. The codes and documentation can be found in “code” and “docs” folders.
Related research article	none

## 1 Value of the Data

• The data offers a rare and structured representation of the German mechanical engineering industry during the pre-World War II era, providing valuable insight into economic history that is otherwise fragmented in unstructured sources.
• Researchers in history, economics, and industrial studies can reuse this dataset to explore the industrial landscape of early 20th-century Germany.
• The MBI-KG is semantically enriched and structured data following the FAIR principles, making it interoperable with other datasets. This allows researchers to integrate these data into broader studies involving economic history, industrial networks, or sociopolitical analyses across different time periods and regions.

## 2 Background

A recent overview of the most important sources and data available for the German capital market highlights their importance and limitations, illustrates practical applications, and shows their value for financial analysts and historians [6]. It is evident that economic studies on the capital endowment and other factors of corporate success have so far focused primarily on stock corporations, as detailed information is available on these due to their disclosure obligations. What is lacking is systematic data on the development of other types of companies, such as partnerships or limited liability companies [7].

The original motivation for creating the MBI-KG dataset was the need to preserve and make accessible the detailed economic data presented in Herbert Patschan's 1937 book “Die Maschinen-Industrie im Deutschen Reich”. This book provides a complete overview of all companies in the German mechanical engineering industry during a crucial historical period. This makes it possible to systematically explore the significance of different legal forms of company in a key German industry - and thus avoids overestimating the role of joint stock companies. The available information also makes it possible to precisely determine the geographical distribution of German mechanical engineering companies. However, the data was locked in a non-digital format, limiting its accessibility and usability for modern research methods.

The project aimed to digitize this historical resource and enhance its utility through the application of Optical Character Recognition (OCR) and semantic enrichment processes. By structuring the data into a knowledge graph, it became more accessible and interoperable, in accordance with contemporary data sharing and reuse standards. This methodological approach not only preserves the historical data but also enables new avenues for research across various disciplines, such as economic history, digital humanities, and data science.

## 3 Data Description

**Data structure.** The MBI-KG reproducible package is organized into a structured hierarchy of folders and files, each containing different aspects of this project. Below is an overview:

MBI-KG/ is a root directory

**Table:** 

│—— docs/ ——> documentation for this project
│ │—— talks/ ——> two presentations with details on data processing and knowledge graph creation
│ │—— sparql_examples/ ——> examples for SPARQL queries
│ └—— README_docs.md ——> a documentation for docs-folder
│—— data/ ——> raw, processed, structured, and enriched data
│ │—— structured_data/ ——> structured data extracted via book2entities.py script
│ │—— scanned_images/ ——> scanned images in JPEG format with 400 dpi resolution
│ │—— ocr_output/ ——> OCR output files in PAGE-XML format
│ │—— models/ ——> OCR and layout-recognition models
│ │—— kg-dataset/ ——> data exported from the MBI-KG in CSV, RDF (ttl), JSON, and NDJSON formats
│ └—— README_data.md ——> a documentation for the data folder
│—— code/ ——> a folder with codes used in this project
│ │—— semantify.py ——> a script for data enriching and semantification
│ │—— requirements.txt ——> Python dependencies with fixed versions
│ │—— entities2kg.py ——> a script for data upload into a Wikibase knowledge graph
│ │—— data_properties.py ——> a script for creating descriptive statistics for properties
│ │—— create_bulk_files_cli.py ——> a script for creating bulk files in TTL and JSON formats using the command line interface and can be only used by admins of the knowledge graph
│ │—— create_bulk_files_api.py ——> a script for creating bulk files in CSV and NDJSON formats using the SPARQL endpoint and can be used by anyone
│ │—— book2entities.py ——> a script for data structuring
│ └—— README_code.md ——> a documentation for the code folder
│—— README.md ——> a README file for the whole project
│—— LICENSE.md ——> licenses for data, code and other content
│—— CONTRIBUTING.md ——> a file describing how to contribute to this project
│—— CODE_OF_CONDUCT.md ——> a code of conduct for this project
└—— CITATION.cff ——> a file providing guidelines for citation

**Data properties.** The MBI-KG contains data about 5150 companies. Table 1 illustrates properties of companies in the bulk files, their identifiers, and counts of the corresponding non-empty values. The properties with capitalized labels correspond to original properties extracted from the book. The properties with non-capitalized labels are additional properties created via semantic enrichment. The property with identifier P2 is Wikidata QID, and the table introduces two more properties which are used in bulk files for Wikidata QIDs of headquarters and legal forms. All properties can be also found via the frontend of the knowledge graph and its SPARQL endpoint.

**Table 1:** The counts of non-empty values of statements with properties (including their English and German labels and their identifiers) extracted from the MBI-KG CSV bulk file.

Property identifier	German property label	English property label	Count of non-empty values
P4	ROH_TEXT	RAW_TEXT	5150
P3	ist_ein	instance of	5150
P5	FILE_SEGMENT	FILE_SEGMENT	5150
P47	Land	country	5150
P6	FABRIKATIONSPROGRAMM	PRODUCTION_PLAN	4190
P8	FERNRUF	TELEPHONE_NUMBER	4101
P10	BANKVERBINDUNGEN	BANK_ACCOUNTS	4028
P46	Gründung	inception	4021
P7	POSTSCHECKKONTO	POSTAL_GIRO_ACCOUNT	3795
P9	DRAHTANSCHRIFT	TELEGRAPHIC_ADDRESS	3525
P29	GESCHÄFTSJAHR	FINANCIAL_YEAR	3497
P44	STRASSE	STREET	3227
P43	STADT	CITY	3073
part of P48	HauptstandortLänge	headquarters longitude	2430
part of P48	HauptstandortBreite	headquarters latitude	2430
P48	Hauptstandort	headquarters location	2430
same as P2	HauptstandortWikidataQIDs	headquarters Wikidata QID	2430
P11	ANLAGEN	FACILITIES	2389
P12	INHABER	OWNER	1875
P13	GRUNDBESITZ	PROPERTY	1778
P42	RECHTSFORM	LEGAL_FORM	1718
P45	Rechtsformen	legal form	1715
same as P2	RechtsformWikidataQIDs	legal form Wikidata QID	1715
P16	GEFOLGSCHAFT	FOLLOWERS	1347
P24	KAPITAL	CAPITAL	1328
P14	ANGABEN	STATEMENTS	1283
P15	PROKURISTEN	AUTHORIZED_SIGNATORIES	1245
P17	EIGENE_VERTRETUNGEN	REPRESENTATIONS	1108
P18	GESCHÄFTSFÜHRER	MANAGING_DIRECTOR	1062
P20	SIEHE	SEE	894
P49	im_Eigentum_von	owned by	648
P21	AUFSICHTSRAT	SUPERVISORY_BOARD	474
P22	ANTEILSEIGNER	SHAREHOLDERS	465
P23	VORSTAND	MANAGEMENT_BOARD	436
P54	hat_Prokurist	has authorized signatory	425
P27	NUTZFLÄCHE	USABLE_SPACE	240
P25	TOCHTERGESELLSCHAFTEN	SUBSIDIARIES	193
P26	AKTIONÄRE	STOCKHOLDER	190
P28	GESELLSCHAFTER	PARTNER	134
P30	FIRMA_GEHÖRT	COMPANY_OWNED_BY	81
P31	BETEILIGUNGEN	SHARES	34
P32	KOMPLEMENTÄRE	GENERAL_PARTNERS	27
P33	SPEZIALITÄT	SPECIALIZATION	19
P35	GESCHÄFTSINHABER_FÜHRER	OWNER_MANAGER	18
P34	BEVOLLMÄCHTIGTE	AUTHORISED_REPRESENTATIVE	18
P36	NIEDERLASSUNGEN	BRANCHES	16
P37	UMSATZ	REVENUE	8
P38	VERTRÄGE	CONTRACTS	7
P39	VERKAUFSBÜRO	SALES_OFFICE	6
P40	KOMMANDITISTEN	LIMITED_PARTNERS	5
P41	FABRIKATIONSANLAGEN	MANUFACTURING_PLANTS	4

## 4 Experimental Design, Materials and Methods

### 4.1 Experimental design

The project was organized into a multi-step process that included digitization, text extraction, data structuring, and semantic enrichment to produce a machine-readable knowledge graph. The data were processed to ensure high accuracy and consistency, allowing for enhanced data integration and reuse.

### 4.2 Instruments, software and tools

1. Scanner:
  a. Microbox book2net ultra
2. OCR software:
  a. eScriptorium [3]
  b. Kraken [4]
3. Data structuring software:
  a. blatt [5]
4. Semantic enrichment tools:
  a. KG-enricher [8]
5. Knowledge graph software:
  a. Wikibase [9]
  b. WikidataIntegrator [10]

### 4.3 Methods

**Fig. 1:**
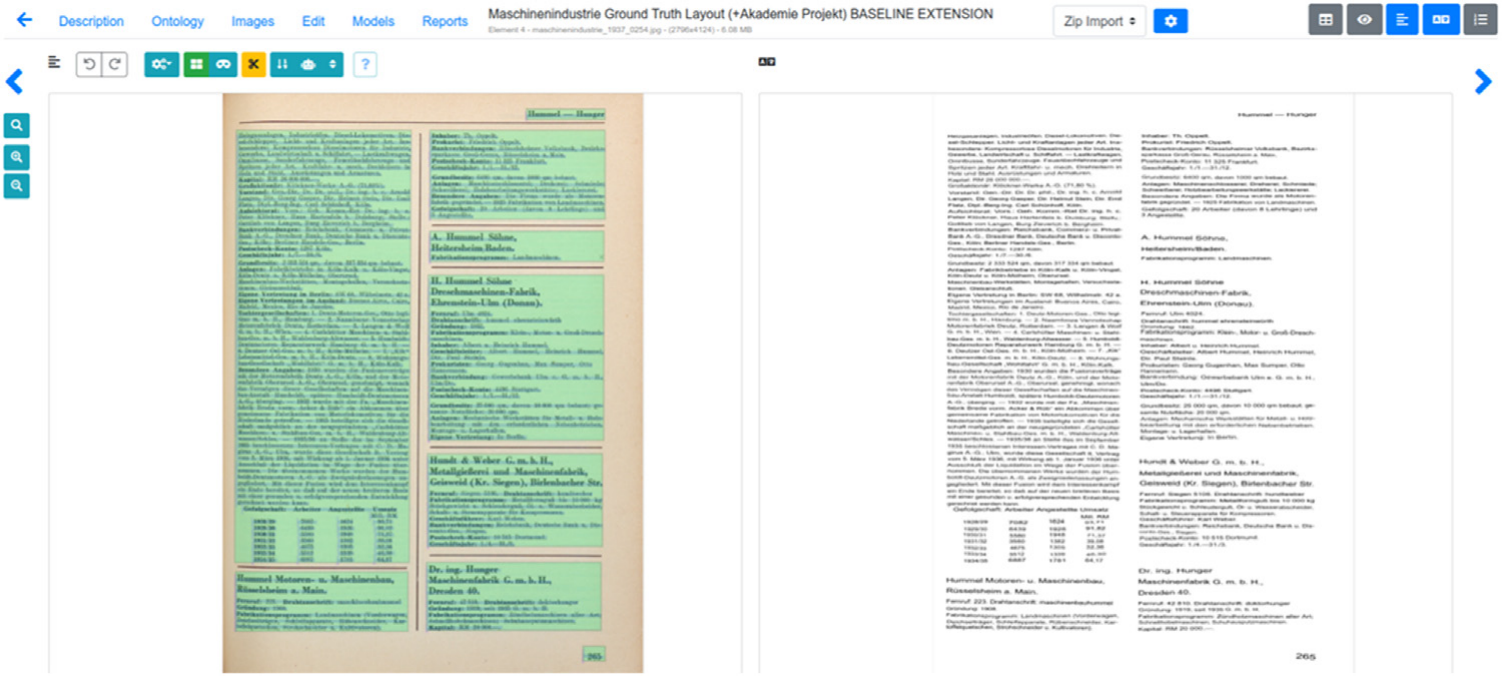
Web-based transcription platform eScriptorium displaying a page from “Die Maschinen-Industrie im Deutschen Reich”.

1. Digitization:
  The original book was scanned using a book2net ultra by Microbox scanner, producing images in JPEG format with a resolution of 400 dpi.
2. OCR processing:
  Text recognition was performed using the Kraken OCR engine, which is integrated with the web-based transcription platform eScriptorium (Fig. 1).

**Fig. 2:**
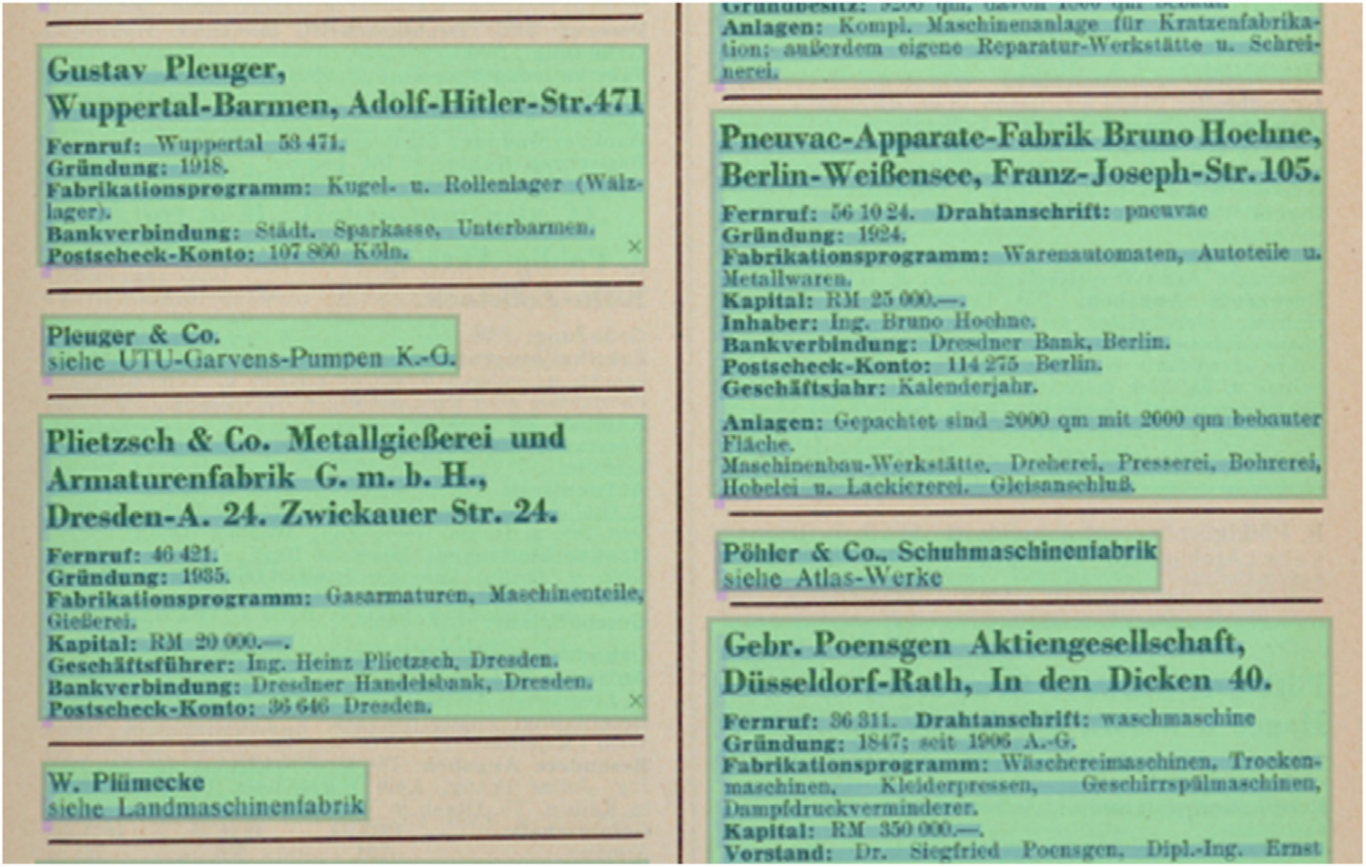
Two-column layout showing individual company entries, with each entry featuring detailed information on a specific company.

This step required customization of the OCR workflow by training specific models tailored to the unique fonts and layouts found in the historical textual material. To ensure high quality and compatibility with other training data, the second level of the OCR-D Ground Truth Guidelines (https://ocr-d.de/en/gt-guidelines/trans) was selected as the underlying guidelines for creating the dataset. The biggest obstacle to improving the OCR accuracy, and thus producing high quality transcriptions, was the two-column layout of the page, with each column containing several individual company entries. Each entry is placed in its own layout region divided by separators (Fig. 2). A dataset of 47 pages for layout segmentation and 26 pages for fine-tuning text recognition was created to achieve the desired 99.3 % accuracy for research data.

The results of the OCR workflow are available in the PAGE XML format, which preserves not only the textual content, but also the structural and layout information of the documents. This format ensures compatibility and interoperability with further post-processing tasks, such as layout segmentation and transcription corrections or additional model training, thus improving the accuracy and usability of the OCR results.

**Fig. 3:**
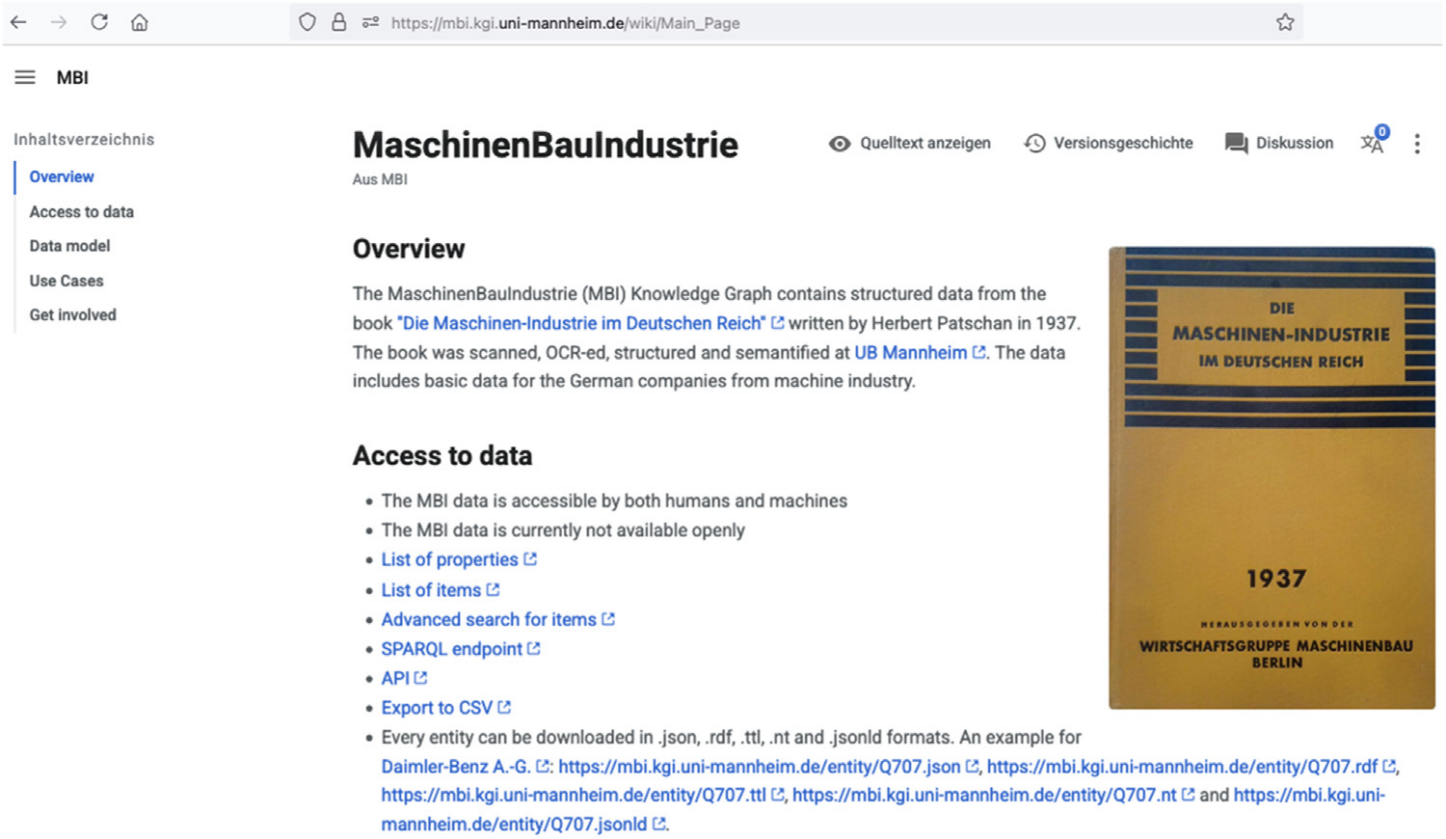
The frontend of the MBI knowledge graph describing data access options, data model, use cases, and ways to be involved in this project.

**Fig. 4:**
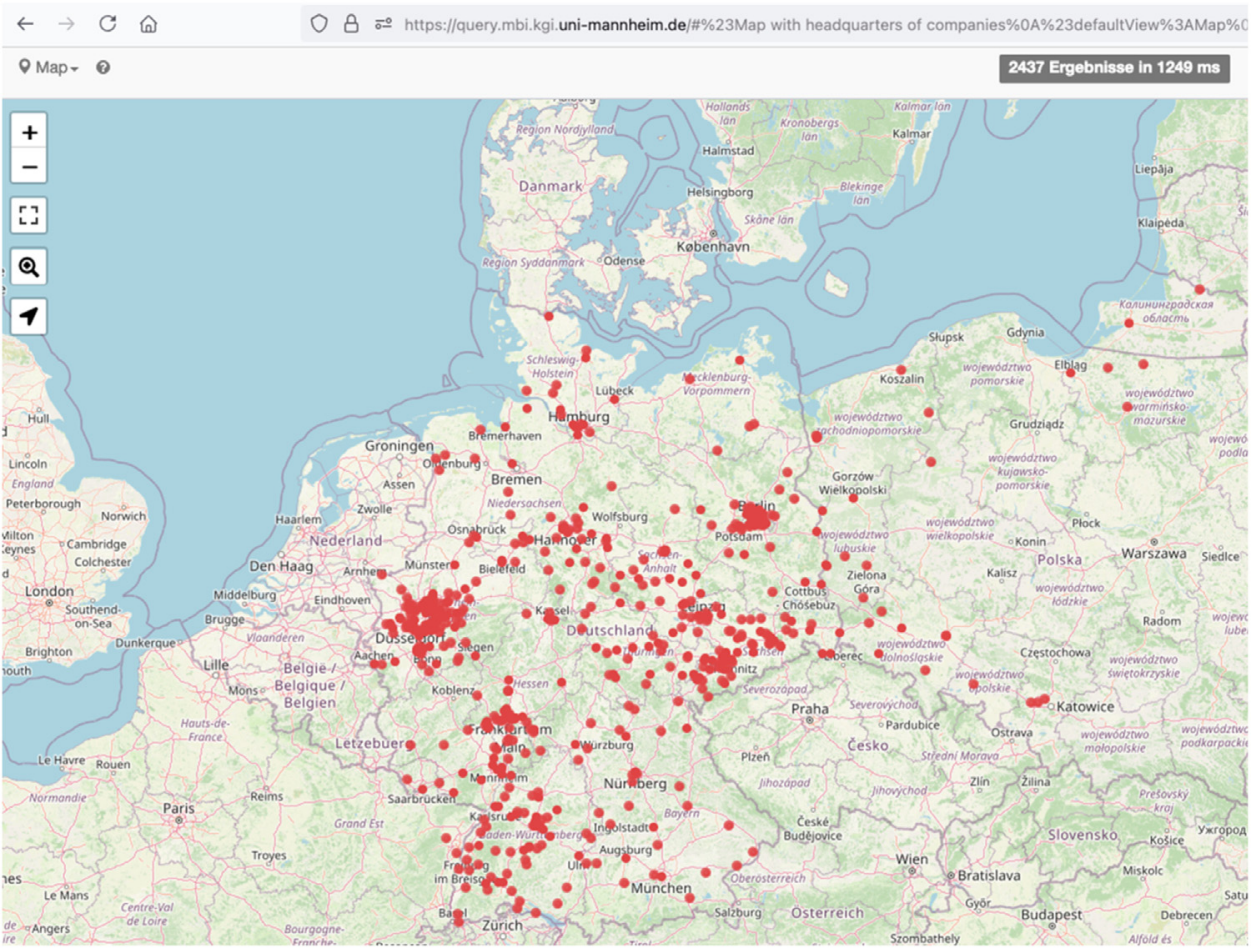
The SPARQL query service of the MBI knowledge graph illustrating the headquarters of companies using geographic coordinates of cities from Wikidata.

3. Data structuring: The unstructured text extracted by OCR was processed using the custom script “book2entities” (https://github.com/UB-Mannheim/blatt/blob/main/projects/MI1937/book2entities.py) based on the Python tool "blatt". Our script parsed the PAGE-XML files, split texts into segments with separate entities, removed headers and page numbers, merged bottom-left with upper-right text regions, merged segments from consequent pages, removed hyphens from OCR-ed strings (using all hyphens described in the OCR-D guidelines for hyphenation https://ocr-d.de/en/gt-guidelines/trans/trSilbentrennung.html), structured the unstructured and unhyphenated texts using colons as separators, performed quality checks, created entities for companies with various structured properties, sorted and grouped properties, merged values within the groups of manually curated properties, and saved structured data as a CSV-file into data/structured_data/MBI_1937_structured.csv.
4. Data upload to a knowledge graph: The structured data in a CSV-file was uploaded to a Wikibase instance using the script entities2kg.py based on the WikidataIntegrator Python library.
5. Semantic enrichment: The structured data in the knowledge graph was further semantically enriched using the script semantify.py based on the KG-enricher tool. This tool links input strings to companies, people, and geographic entities in the Wikidata knowledge graph and returns enriched structured information. Geographic strings were further validated against modern and historical German boundaries using the CShapes 2.0 dataset [11]. We also enriched cities with their geographic coordinates and created legal forms as entities. Manual quality control was performed via the graphical user interface of the knowledge graph (Fig. 3). Fig. 4 shows a frontend of the SPARQL query service illustrating the headquarters of companies using geographic coordinates of cities from Wikidata. We created bulk files of the knowledge graph using two scripts. The script create_bulk_files_api.py sends queries the MBI-KG SPARQL endpoint and saves bulk dataset in CSV and NDJSON formats. It can be reused by everyone to get up-to-date bulk files from the knowledge graph. The second script create_bulk_files_cli.sh is a command line tool for admins of the knowledge graph and based on the internal helper php-scripts of Wikibase for creating bulk files in ttl and JSON formats.

## Limitations

The MBI-KG dataset has a few limitations. The OCR-process introduces errors. The historical nature of the source material, including the specific typefaces and the quality of the original print, could result in inaccuracies in text extraction, even with customized OCR models. During the data structuring process, some complexities and ambiguities in the raw data might not have been perfectly captured, leading to potential inconsistencies in the structured dataset. The semantic enrichment process using the kg-enricher tool relies on external knowledge graphs such as Wikidata, which may introduce biases or gaps, especially when linking historical entities not fully represented in modern datasets. The validation of geographic entities against the CShapes 2.0 Dataset may not fully account for historical territorial changes, potentially affecting the accuracy of geographic data. Some of these limitations can be addressed manually through the user interface of MBI-KG. If you encounter errors in the data, please open an issue on GitHub at https://github.com/UB-Mannheim/MBI-KG or contact us via email.

## Ethics Statement

The authors confirm that they have read and followed the ethical requirements for publication in Data in Brief. This work does not involve human subjects, animal experiments, or any data collected from social media platforms.

## CRediT authorship contribution statement

**Renat Shigapov:** Writing – original draft, Methodology, Software, Validation, Data curation. **Thomas Schmidt:** Writing – original draft, Methodology, Software, Validation, Data curation. **Jan Kamlah:** Writing – original draft, Methodology, Software, Validation, Data curation. **Irene Schumm:** Writing – review & editing, Supervision, Project administration, Funding acquisition. **Jochen Streb:** Writing – review & editing, Supervision, Project administration. **Sibylle Lehmann-Hasemeyer:** Writing – review & editing, Supervision, Project administration.

## Data Availability

MADATAMBI-KG: Replication package for a knowledge graph of structured and linked economic research data extracted from the 1937 book “Die Maschinen-Industrie im Deutschen Reich” (Original data)
